# Complementary Role of Combined Indirect and Direct Cardiac Sympathetic (Hyper)Activity Assessment in Patients with Heart Failure by Spectral Analysis of Heart Rate Variability and Nuclear Imaging: Possible Application in the Evaluation of Exercise Training Effects

**DOI:** 10.3390/jcdd9060181

**Published:** 2022-06-05

**Authors:** Ferdinando Iellamo, Marco Alfonso Perrone, Andrea Cimini, Giuseppe Caminiti, Agostino Chiaravalloti, Attilio Parisi, Orazio Schillaci

**Affiliations:** 1 Department of Cardiology and Sports Medicine, University of Rome Tor Vergata, 00133 Rome, Italy; iellamo@uniroma2.it; 2 Cardiology Rehabilitation Unit, Istituto di Ricovero e Cura a Carattere Scientifico IRCCS San Raffaele Pisana, 00163 Rome, Italy; giuseppe.caminiti@sanraffaele.it; 3 Department of Biomedicine and Prevention, University of Rome Tor Vergata, 00133 Rome, Italy; andreacimini86@yahoo.it (A.C.); agostino.chiaravalloti@uniroma2.it (A.C.); orazio.schillaci@uniroma2.it (O.S.); 4 Department of Human Science and Promotion of Quality of Life, San Raffaele Open University, 00163 Rome, Italy; 5 Nuclear Medicine Section, IRCCS Neuromed, 86077 Pozzilli, Italy; 6 Department of Movement, Human and Health Sciences, Division of Health Sciences, University of Rome Foro Italico, 00135 Rome, Italy; attilio.parisi@uniroma4.it

**Keywords:** heart failure, exercise training, heart rate variability, MIBG, cardiac autonomic regulation

## Abstract

In chronic heart failure (CHF), abnormalities in cardiac autonomic control, characterized by sympathetic overactivity, contribute to the progression of the disease and are associated with an unfavorable prognosis. Assessing cardiac autonomic status is clinically important in the management of patients with CHF. To this aim, heart rate variability (HRV) analysis has been extensively used as a non-invasive tool for assessing cardiac autonomic regulation, and has been shown to predict the clinical outcome in patients with CHF. Adrenergic nerve activity has also been estimated using iodine-123 (I-123) metaiodobenzylguanidine (MIBG), a noradrenaline analogue. MIBG is an analogue of norepinephrine sharing the same cellular mechanism of uptake, storage, and release in presynaptic sympathetic neurons. As an innervation tracer, 123I-MIBG allows for the evaluation of cardiac sympathetic neuronal function. Cardiac MIBG imaging has also been reported to predict a poor clinical outcome in CHF. MIBG provides direct information on the function of the presynaptic sympathetic nerve endings, whereas HRV, which depends on postsynaptic signal transduction, reflects the end-organ response of the sinus node. The aim of this brief review is to provide the reader with some basic concepts regarding the spectral analysis of HRV and MIBG, highlighting what is known about their respective roles in detecting cardiac sympathetic hyperactivity in CHF and, in perspective, their possible combined use in assessing non-pharmacological treatments in patients with CHF and reduced ejection fraction, with a particular focus on the effects of exercise training.

## 1. Introduction

In chronic heart failure (CHF), abnormalities in cardiac autonomic control, characterized by sympathetic overactivity and parasympathetic withdrawal [1], contribute to the progression of the disease through a vicious circle by which the initial compensatory increase in neurohumoral activation leads, in time, to worsening of functional status [1,2,3]. This is associated with an unfavorable prognosis [2,3]. Therefore, assessing cardiac autonomic status is clinically important in the management of patients with CHF. Heart rate variability (HRV) analysis is widely used as a non-invasive tool for the assessment of cardiac autonomic regulation [4], and has been shown to predict the clinical outcome in patients with CHF [5,6,7,8,9].

Adrenergic nerve activity has also been estimated using iodine-123 (I-123) metaiodobenzylguanidine (MIBG), a noradrenaline analogue [10,11]. This radiopharmaceutical is an analogue of norepinephrine (NE), with the same cellular mechanism of uptake, storage, and release in presynaptic sympathetic neurons [11]. As an innervation tracer, 123I-MIBG allows for the evaluation of cardiac sympathetic neuronal function. Cardiac MIBG imaging has also been reported to predict a poor clinical outcome in CHF [12,13,14,15,16]. Although it could be envisaged that there is a close relation between the two techniques, few studies have examined the relationship between parameters provided by cardiac MIBG imaging and HRV analysis in patients with CHF [16,17,18]. Indeed, the possible new information on sympathetic cardiac control that could ensue from the simultaneous assessment of both pre-and post-synaptic sympathetic activity in CHF is still scant.

Cardiac MIBG imaging provides direct information on the function and integrity of the presynaptic sympathetic nerve endings [10,19,20]. On the other hand, HRV, which depends on postsynaptic signal transduction, reflects the end-organ response of the sinus node. In conditions characterized by marked, persistent sympathetic activation, as observed in chronic HF, the sinus node may drastically diminish its responsiveness to neural inputs. The HRV “indirectly” reflects the end-organ response of the sinus node to both sympathetic and parasympathetic nerve discharge, whereas cardiac MIBG imaging “directly” reflects sympathetic nerve function only.

The aim of this brief review is to provide the reader with some basic concepts on the spectral analysis of HRV and MIBG, highlighting what is known about their respective roles in detecting cardiac sympathetic hyperactivity in CHF, and their possible combined use in assessing the effect of both pharmacological and non-pharmacological treatments in patients with CHF and reduced ejection fraction (HFrEF), with a particular focus on the effects of exercise training.

## 2. Spectral Analysis of HRV in Patients with CHF

Patients with CHF are at risk for arrhythmias. This is a consequence of impaired autonomic control of the heart, characterized by enhanced sympathetic and decreased vagal activities of the heart [21,22,23]. This condition translates to a reduced HRV that, in turn, is linked to a greater risk of arrhythmias [23,24]. Reduced HRV parallels a deterioration in CHF status [24] and worsens the prognosis [24,25,26]. Exercise training has been shown to be capable of improving HRV in patients with CHF [27,28], and is currently highly recommended by guidelines all around the world [29,30]. However, the optimal “dose” of exercise, defined in terms of volume and intensity, required to achieve improvements in functional and prognostic parameters, still remains a crucial unanswered issue. Indeed, defining the optimal dose of exercise to maximize health outcomes is now considered a priority. The best format of exercise (e.g., interval vs. continuous), the differences in individual internal training loads, and the limits of HR-derived methods in planning exercise in CHF are also debated. Exercise training guided by HR or HR reserve (HRR), as is the current norm, can be limited in CHF patients because of chronotropic incompetence and beta-blocker treatment.

**Figure 1:**
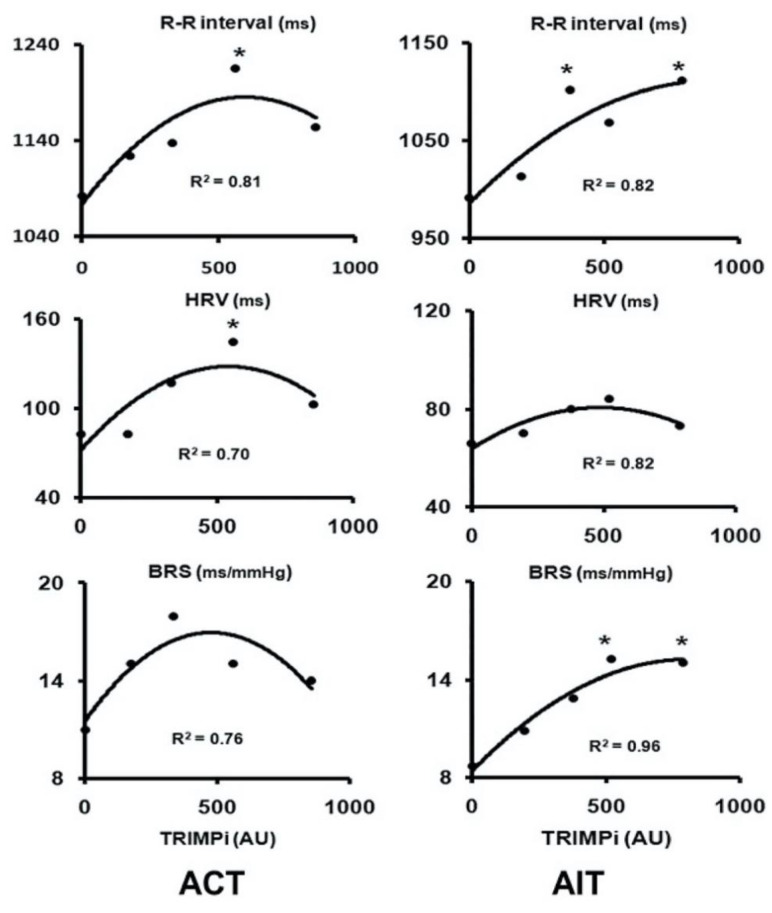
Dose–response relationship between weekly TRIMPi and autonomic cardiovascular parameters during aerobic continuous training (**left panel**) and aerobic interval training (**right panel**). * *p* < 0.05 versus pretraining baseline values. ACT: aerobic continuous training. AIT: aerobic interval training. HRV: heart rate variability. BRS: baroreflex sensitivity. Reproduced with permission from Iellamo et al. [35].

Iellamo and co-workers [31] recently performed a series of studies addressing (1) the relationship between the dose of exercise and autonomic control of HR and (2) the effects of different exercise formats on HRV response and hemodynamic adaptations in patients with chronic heart failure. In these studies [31,32,33], the training stimulus was quantified by using an individualized training methodology. This relatively new methodology, referred to as the TRaining IMPulses methodology (TRIMPi), is a method implemented by Manzi et al. [34] that uses a simple algorithm based on the exponential relationship between the individual HR reserve and blood lactate production during incremental exercise. The methodology takes into account both the external (i.e., energy expenditure) and internal training loads and makes it possible to integrate the volume and intensity of exercise (e.g., the “dose”) in a single term expressed in arbitrary units [34]. The effects of exercise training (both continuous and interval training) on HRV and RR interval (and baroreflex sensitivity (BRS)) are reported in Figure 1.

HRV and BRS, as well as RR interval, increased significantly with both training protocols and were very highly correlated to the dose of exercise with a second-order regression model (R^2^ ranged from 0.75 to 0.96; *p* < 0.001), resembling a bell-shaped curve in the aerobic continuous training group and an asymptotic-shaped curve in the aerobic interval training group, respectively. Peak VO_2_ also increased significantly (*p* < 0.05), without significant differences between the two training protocols. The novel findings of this investigation were as follows: (1) exercise training improves HRV and BRS in CHF patients undergoing beta-blocker treatment; (2) HRV and BRS individual adaptations to exercise training are dose related in a nonlinear fashion; and (3) higher doses of exercise training do not necessarily lead to a greater improvement in HRV and BRS. The above results imply that a moderate dose of exercise, corresponding to exercising at 55% to 60% of HRR for 40 to 45 min four times a week, is sufficient to achieve substantial improvements in HRV and BRS. No substantial improvements in exercise performance occurred with more vigorous activity; rather, a reduction in HRV and BRS was observed (Figure 1). This finding is clinically relevant, as more vigorous exercises, with the attendant increase in sympathetic activity, might pose a risk for arrhythmic episodes in a high-risk population such as CHF patients. It does appear that in patients with CHF, the potential benefit of increasing exercise performance by increasing the training load from moderate to higher doses of exercise should be weighed against the increase in cardiac sympathetic modulation with the possible increased risk of adverse events.

Interestingly, a prior study from the same group [34], performed in marathon runners and employing spectral analysis of HRV, demonstrated the same dose–response relationship between the training load and HRV parameters, with a decrease at lower loads and an increase at higher loads in the low-frequency (LF) component of HRV, an indirect index of cardiac sympathetic modulation. This finding confirmed, on an individual basis, a study by Iellamo et al. in elite class rowers [32], which showed conversion from vagal to sympathetic predominance from lower to higher training loads [35]. 

These findings have clear clinical implications in planning exercise training programs in patients, like CHF, who feature an already high baseline sympathetic activity. 

The interested reader is referred to Appendix A for a more in-depth discussion on the spectral analysis of HRV.

## 3. Nuclear Imaging with 123I-Metaiodobenzylguanidine in Patients with CHF

**Figure 2:**
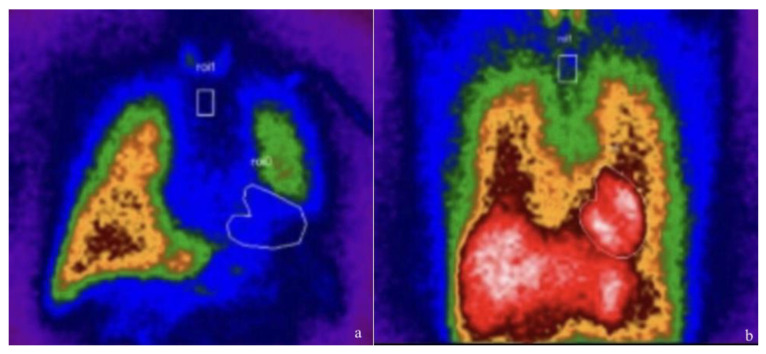
123I-metaiodobenzylguanidine (123I-MIBG) cardiac scintigraphy (planar images at 4 h) in a patient with heart failure ((**a**), absent myocardial uptake of the radiopharmaceutical) and in a healthy patient ((**b**), normal myocardial uptake of the tracer).

A more direct measure of sympathetic activity at the heart level is scintigraphy with the 123I-metaiodobenzylguanidine (MIBG) tracer, which, at present, is the most widely used imaging agent for studying the causes and effects of cardiac sympathetic hyperactivity. MIBG is an analogue of norepinephrine (NE), with the same cellular mechanism of uptake, storage, and release in presynaptic sympathetic neurons [36]. The uptake of 123I-MIBG into neurons is mainly achieved through the uptake-1 mechanism, a homeostatic system responsible for the reuptake of NE. Unlike NE, MIBG is not metabolized, allowing it to be imaged. By using this technique, myocardial uptake of NE and its distribution can be visually assessed (Figure 2).

**Figure 3:**
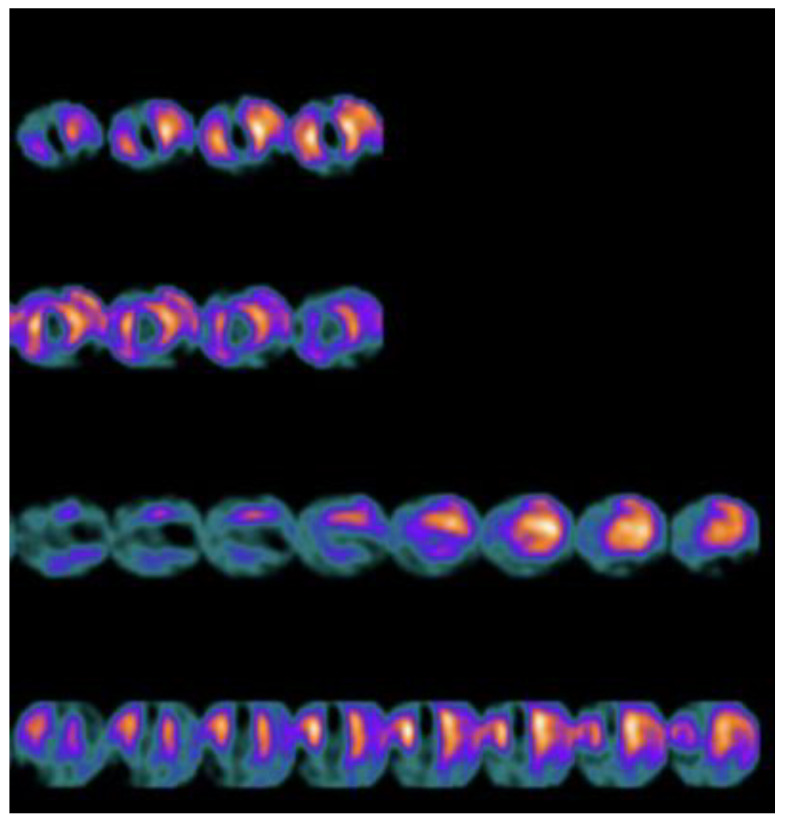
123I-meta-iodobenzylguanidine (123I-MIBG) single-photon emission computed tomography (SPECT) in a patient with previous myocardial infarction of the apex, with involvement of the anterior and the posterior wall (absent uptake of the radiopharmaceutical in these sites).

MIBG uptake is semiquantified by calculating the heart-to-mediastinum ratio (HMR) after drawing regions of interest over the heart and mediastinum [37]. This approach provides an index of cardiac sympathetic activity [38]. By comparing early and delayed activities (15 min vs. 4 h after MIBG injection), the MIBG wash-out (WO) rate from the myocardium can be derived, providing a parameter that reflects the retention of NE by sympathetic neurons [39]. It has been shown that the uptake of 123I-MIBG is significantly reduced in areas of myocardial infarction [40], as well as in areas with chronic ischemia [41,42]. Moreover, it is important to underline that cardiac 123I-MIBG single-photon emission computed tomography (SPECT) may provide useful information regarding the size, accurate localization, and severity of innervation abnormalities [43] (Figure 3).

Concordance between the extent of 123I-MIBG defect during rest and perfusion defect during exercise has been shown in patients with coronary artery disease, which is the main cause of CHF. This concordance suggests that resting imaging with 123I-MIBG combined with resting myocardial perfusion imaging (MPI) may be useful to assess the cardiac sympathetic (hyper)activity that characterizes CHF. 123I-MIBG might be used for risk stratification and prognosis too, since it has been shown that the late (reduced) HMR is an independent predictor of mortality, with late HMR being the best predictor of event-free survival [37]. A reduced late HMR has been reported as the most powerful predictor of cardiac mortality in patients with CHF [44,45].

These findings regarding risk stratification and prognosis by MIBG appear to resemble those reported with the indirect technique of HRV analysis. The prognostic value of cardiac 123I-MIBG imaging, together with that of time and frequency domain parameters of HRV, has been prospectively evaluated in patients with mild-to-moderate CHF [46], showing an association between late HMR, wash-out (WO) rate and low-frequency power of HRV with cardiac events at follow-up.

123I-MIBG imaging has also been utilized to evaluate the effect of various drugs and their combinations on cardiac sympathetic functioning. In these studies [47,48,49,50,51], an increase in HRM and a decrease in WO rate have consistently been reported, being linked with improvements in symptoms, function, and survival.

It is worthwhile to mention that MIBG has been successfully employed to evaluate not only the cardiac sympathetic response and remodeling to various drugs currently used in the management of CHF [49,50,51], but also to assess the response to exercise training.

Exercise training (ET) would lead to a lower resting cardiac sympathetic stimulus [52,53] and may be associated with improved MIBG parameters. Agostini et al. [54] reported a significant increase in myocardial MIBG uptake, along with an improvement in functional capacity, after 6 months of ET in class II-III CHF patients. These results are in accordance with studies reporting positive effects of ET on HRV parameters in CHF patients, even those undergoing beta-blocker treatment [31,33]. Some studies, however, found no effect of ET on MIBG in this patient population [55]. Differences in study populations and sample sizes, types and durations of ET could have all contributed to this discrepancy. To the best of our knowledge, only a few studies have investigated CHF patients with both MIBG and HRV techniques. Yamada et al. [46] investigated the prognostic value of MIBG imaging and HRV parameters in patients with mild-to-moderate CHF, but did not report on their possible link. Similarly, Tamaki et al. [56] reported a significant prognostic value of cardiac MIBG imaging for sudden death in CHF, not observed for HRV, but here again, the link between MIBG and HRV parameters was not investigated as part of the study, which focused substantially on the predictive role of MIGB in sudden death in CHF.

Importantly, no study addressed the same individual patients’ MIBG and HRV responses to exercise training and their possible link. Exploring the whole sympathetic functioning response, i.e., both pre- and postsynaptic, in relation to exercise training by combining MIBG and HRV assessment, might prove to be a meaningful tool to gain a better understanding of the mechanism(s) underlying the autonomic benefits of exercise training in CHF, with the obvious clinical implications.

It should be outlined that all the studies performed so far for assessing HRV in association with MIBG suffer from a strong methodological limitation. In fact, all the studies investigated HRV using a 24 h ECG recording. HRV analysis on 24 h ECG ambulatory recording carries many drawbacks. In long-term recordings, the HF and LF components account for only approximately 5% of the total power, the remaining being constituted by very-low (VLF) and ultra-low frequency (ULF) components. The consequence of this methodological issue is exemplified by the study of Yamada et al. [46], who reported a prognostic role of cardiac events in CHF for VLP, but not for other HRV spectral components. The physiological explanation of the VLF component is poorly defined, and the existence of a specific physiological process attributable to these heart period changes is still under scrutiny. Another fundamental issue in the spectral analysis of HRV is the “stationarity” of the ECG signal. The problem of “stationarity” is frequently discussed with long-term recordings. Stationarity (or quasi-stationarity) of the signal is a prerequisite for the power spectral analysis of HRV. If the modulations are not stable, the interpretation of the results of the frequency analysis is less well defined. In particular, physiological mechanisms of heart period modulations responsible for LF power components (and HF components as well) cannot be considered stationary during the 24 h period. To ascribe individual spectral components to well-defined physiological mechanisms, such mechanisms modulating the heart rate should not change during the recording. The lower stability of heart rate modulations during long-term recordings makes the results of frequency analysis less easily interpretable. In 24 h recordings, autonomic modulation of RR interval is influenced by several factors related to physical activity, changing postures, emotional circumstances, awake/sleep periods, environment, etc., which makes HRV analysis unpredictable. This obscures the detailed information about autonomic modulation of RR intervals that, instead, is available in shorter recordings (e.g., 510 min) obtained in stationary conditions. To date, short-term HR recordings in association with MIBG nuclear imaging has not been conducted.

Finally, another factor that is further confounding is the different times at which the MIBG and the HRV were actually performed (even more than one day apart). In fact, HRV may vary in a given individual within the same day, and this could affect the interpretation of the frequency components of HRV, especially if used to infer possible links with other techniques exploring autonomic cardiac regulation, such as MIGB nuclear imaging.

## 4. Possible Applications and Perspectives of MIBG Nuclear Imaging

While routine clinical use of 123I-MIBG imaging for monitoring heart disease status or treatment response is unlikely, because of economic issues, in perspective, imaging of the cardiac sympathetic system combined with simultaneously assessed (i.e., at 15 min and at four hours after an iodine-123 metaiodobenzylguanidine injection to coincide with early and late imaging) autonomic functioning by spectral analysis of HRV could help in quantifying the functional severity of myocardial injury and remodeling associated with CHF, and to address the autonomic, mainly the sympathetic, response to exercise training and the physiological mechanism(s) underlying it. Currently, there are few data in the literature. However, considering nuclear imaging as a reference method for functional imaging of the sympathetic nervous system, in perspective, it could find greater applications in this research topic.

As concerns future perspectives in nuclear medicine for the assessment of cardiac sympathetic function, the potential use of [11C]meta-hydroxy-ephedrine ([11C]mHED), a catecholamine analogue used in positron emission tomography (PET), should be considered [57]; the higher spatial resolution in comparison to 123I-MIBG scintigraphy/SPECT and the short duration of the exam (due to the physical half-life of the radioisotope 11C of about 20 min) make this radiopharmaceutical attractive for this purpose. The studies regarding the application of [11C] mHED PET for the evaluation of cardiac sympathetic innervation have shown promising results in the detection of cardiac areas with impaired sympathetic function [58,59]. 18F-labeled radiopharmaceuticals, such as (18)F-N-[3-bromo-4-(3-fluoro-propoxy)-benzyl]-guanidine (18F LMI1195) [60] or 18F-meta-fluorobenzylguanidine (18F-MFBG, a PET analog of MIBG) [61], may be promising as well, due to the appealing features of the radioisotope 18F (with a physical half-life of about 110 min) in PET imaging. Despite the attractive features of PET tracers, further studies are needed for their use in clinical applications; to date, 123I-MIBG remains the most available radio compound for the assessment of cardiac sympathetic innervation.

## 5. Conclusions

In addition to the spectral analysis of HRV, several papers have shown that nuclear imaging is a reference method for evaluating the autonomic sympathetic system in patients with cardiovascular disease. Data currently present in the literature suggest that nuclear imaging could have an important role in evaluating the effects of exercise training on autonomic cardiovascular regulation in patients with ischemic heart disease and heart failure. Studies also indicate that cardiac nuclear imaging is more suitable for research purposes than for clinical applications. In this context, combining nuclear imaging by MIBG with HRV by spectral analysis might represent a step forward in the functional (as well as clinical) evaluation of the autonomic, mainly sympathetic, cardiac adaptations to exercise training in patients with heart failure.

## Appendix A

Heart rate variability (HRV) is one of the most prominent non-invasive indicators of autonomic nervous system (ANS) functioning. By applying a spectral analysis to RR continuous ECG signal, two main periodic components can be detected: one in the low-frequency (LF) range (0.04–0.15 Hz) and one in the high-frequency (HF) range (0.15–0.4 Hz), which is synchronous with respiration [4]. The LF component (when expressed in normalized units) would represent a marker of cardiac sympathetic modulation of the sinoatrial node, while the HF component would represent an indicator of cardiac parasympathetic modulation. The ratio LF/HF is considered an indicator of the so-called cardiac sympathovagal balance.

In this context, expressing the LF and HF components in normalized units, relative to total power (that is, the overall RR interval variability), is crucial to obtain valuable information about sympathetic and vagal cardiac modulation, due to the high inter-individual variability in RR interval total variance and direct current (DC) noise. Interpretation of the spectral analysis of HRV stimulated scientific debates in the literature [62,63]; nevertheless, several studies have affirmed that the spectral analysis of HRV is a simple way to extract the information embedded in the frequency code characterizing neural cardiovascular regulation. The issue of the physiological meaning of the LF component, in particular, has been addressed by experiments in humans in whom direct recordings of muscle sympathetic nerve activity were performed during various states of autonomic regulation, as produced by graded infusions of vasodilators and vasoconstrictors. The presence of similar, coherent oscillations at low frequency in nerve activity, RR intervals, and systolic arterial pressure (SAP) variability at various levels of induced pressure changes provides support for the use of LF as an index of sympathetic modulation of the sinoatrial node and of LF of SAP as an index of efferent sympathetic vascular modulation [64]. The lack of LF oscillations in the RR interval (and SAP variability as well) in tetraplegic patients, who lack the ability to modulate sympathetic nerve traffic to the heart and vasculature [65], provided further experimental support for the above concept.
